# Stress-Induced Cardiomyopathy Raising Concern for Myocardial Ischemia

**DOI:** 10.7759/cureus.22091

**Published:** 2022-02-10

**Authors:** Arminder Singh, Stephanie Everest, Lam Nguyen, Bradley Casey, Manoj Bhandari

**Affiliations:** 1 Internal Medicine, Cape Fear Valley Medical Center, Fayetteville, USA; 2 Medical School, Campbell University School of Osteopathic Medicine, Lillington, USA; 3 Cardiology, Cape Fear Valley Medical Center, Fayetteville, USA

**Keywords:** takotsubo cardiomyopathy, acute myocardial infarction, acs, cardiomyopathy, stress-induced cardiomyopathy

## Abstract

Stress-induced cardiomyopathy (SIC), or Takotsubo syndrome, is considered a reversible dysfunction of the left ventricle that may mimic an acute coronary syndrome (ACS). Patients may present with chest pain, dyspnea, syncope, or other serious complications including cardiogenic shock, ventricular arrhythmias, and thrombus formation. Diagnostic criteria for SIC include several factors, including electrocardiogram changes, cardiac biomarker elevations, ventricular regional wall abnormalities on echocardiogram, and absence of occlusive coronary disease on coronary angiography. There is no standardized protocol for the treatment of SIC, although the current consensus is that patients should be risk-stratified, managed for complications, and treated with supportive therapy accordingly. In this case study, we present an 85-year-old female who presented with one month of fatigue, lower extremity weakness, and exertional dyspnea with chest pressure. She received a cardiac workup which revealed lab and imaging findings consistent with SIC. She received treatment for SIC, pericarditis, and Clostridium difficile colitis.

## Introduction

Stress-induced cardiomyopathy (SIC), also known as Takotsubo cardiomyopathy, is considered a transient condition characterized by myocardial injury and regional wall motion abnormalities of the left ventricle. SIC is most frequently reported after a significant physical or emotional trigger, and it is colloquially known as “broken heart syndrome.” Clinically, SIC presents with symptoms and exam findings mimicking an acute coronary syndrome (ACS), but SIC is different from ACS by the absence of coronary artery occlusion [1]. It has been estimated that the annual incidence of SIC in the United States is between 50,000-100,000 cases, and approximately 1-2% of patients suspected to have ACS on presentation are ultimately diagnosed with SIC [2]. Rates of SIC are highest in post-menopausal women, especially those with other risk factors for cardiovascular disease and those with a history of certain neurologic or psychiatric disorders such as anxiety disorders [3,4]. Hospitalized patients are at an increased risk of secondary Takotsubo syndrome, which has been reported after a wide variety of acute medical conditions and surgical procedures requiring local or general anesthesia. While SIC is often regarded as a reversible cardiac dysfunction, it is estimated that around one-half of patients with SIC experience serious complications, ranging from acute heart failure, left ventricular outflow obstruction, mitral regurgitation, cardiogenic shock, arrhythmias, thrombus formation, pericardial effusion, and right ventricular involvement. The in-hospital mortality rate for patients with SIC has been estimated to be 4.5%, and five-year recurrence has been reported in 5-22% of patients [2].

Considering the possible complications and the various triggers mentioned, management of SIC can be patient and case-specific. In this report, we discuss the case of an 85-year-old female who presented to the hospital with one month of worsening fatigue, exertional dyspnea, and chest pressure and was ultimately diagnosed and treated for SIC.

## Case presentation

**Figure 1:**
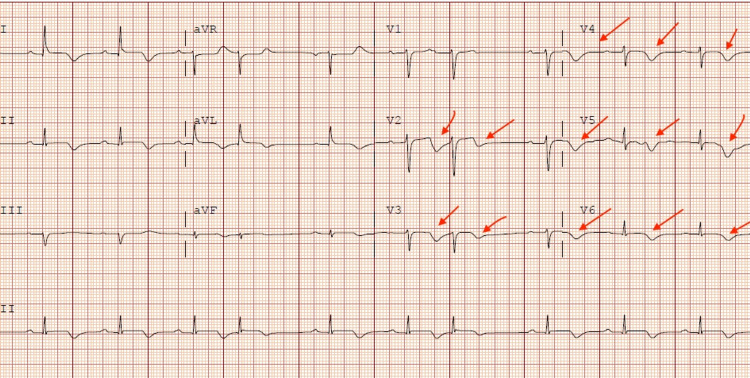
Electrocardiogram (ECG): new t-wave inversions in the anterior lateral leads (red arrow)

An 85-year-old female with a history of chronic kidney disease, hypertension, gout, and previous subdural hematoma presented to the emergency department (ED) after a fall at home. The patient reported difficulty ambulating due to lower extremity weakness and recurrent falls over the past three months. The patient also reported exertional dyspnea, fatigue, and chest pressure, which caused her to feel winded and take multiple pauses when getting herself out of bed. Initial labs were significant for high sensitivity troponin I of 8.840 ng/mL (normal <0.045 ng/mL) and a magnesium level of 1.5 mg/dL (normal 1.6-2.4 mg/dL). Creatinine on presentation was 1.27 mg/dL (normal 0.55-1.30 mg/dL). Her electrocardiogram was concerning for new t-wave inversions in the anterior lateral leads and a prolonged QTc of 538 milliseconds (Figure 1). Initial imaging, including a chest X-ray and CT of the head and spine, were negative for acute findings. Her HEART score was 8. The patient was started on a heparin drip and aspirin per protocol, as well as given 2 grams of IV magnesium, monitored with serial EKGs and troponins, and admitted for further management of suspected non-ST elevated myocardial infarction (NSTEMI). Serial cardiac markers remained elevated, demonstrating a flat troponin trend on the next two troponin levels of 6.600 (normal <0.045 ng/mL) and 5.690 ng/mL (normal <0.045 ng/mL) respectively.

**Figure 2:**
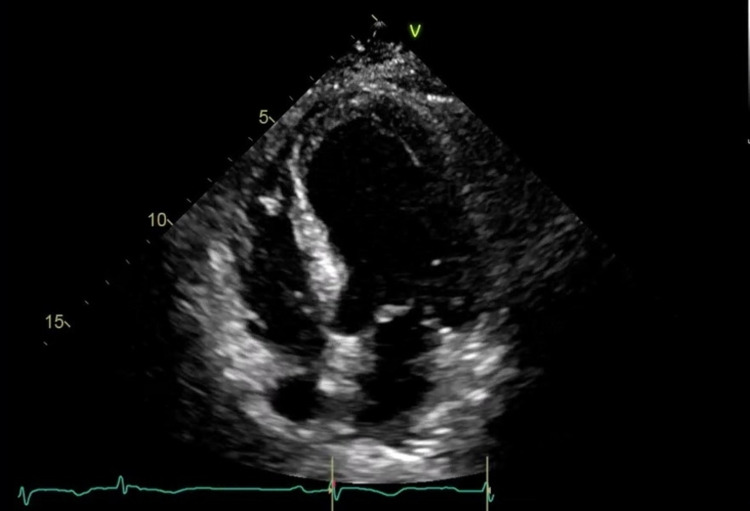
Transthoracic echocardiogram (TTE) demonstrating regional wall abnormalities of the left ventricle during diastole The mid and distal anterior septum, entire apex and mid septum segment are hypokinetic. All other remaining scored segments are normal.

**Figure 3:**
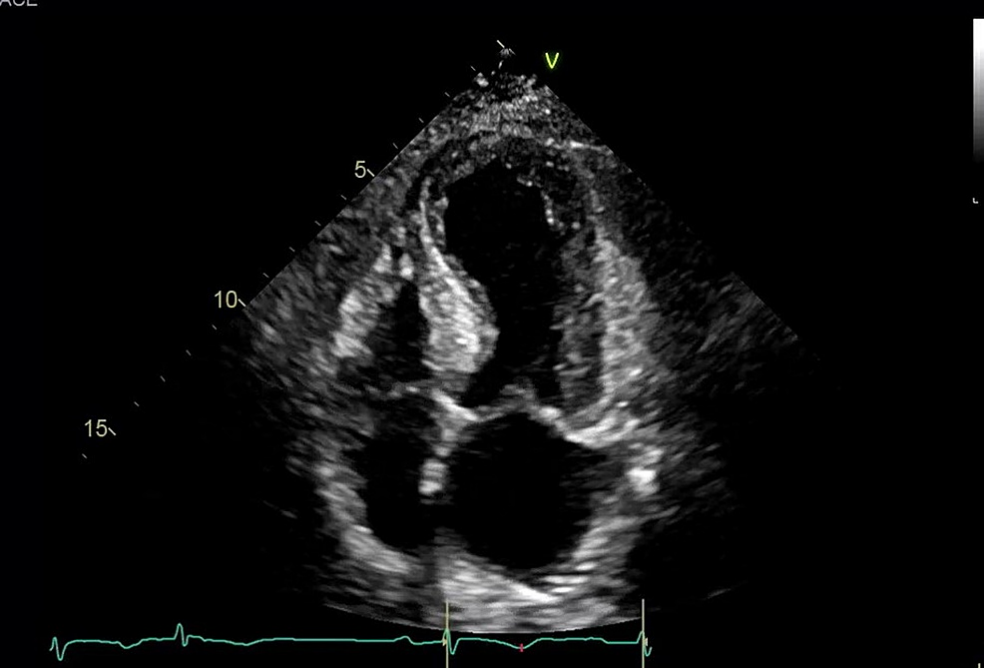
Transthoracic echocardiogram (TTE) demonstrating regional wall abnormalities of the left ventricle during systole The mid and distal anterior septum, entire apex and mid septum segment are hypokinetic. All other remaining scored segments are normal.

Given the patient’s clinical course, elevated cardiac markers, and echocardiogram findings, the differential diagnosis included NSTEMI versus stress-induced (Takotsubo) cardiomyopathy. The patient was started on clopidogrel per protocol with plans for a cardiac catheterization once medically appropriate. Troponin continued to trend down (1.83 ng/mL). The transthoracic echocardiogram revealed apical and diaphragmatic wall segments of the left ventricle were hypokinetic with an ejection fraction of about 50% (Figures 2, 3).

**Figure 4:**
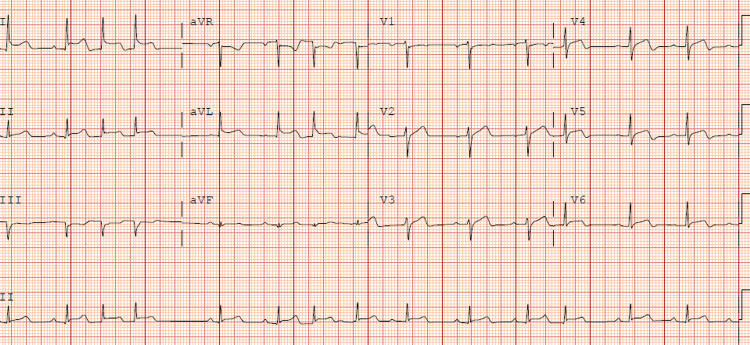
Electrocardiogram (ECG) demonstrates ST elevation prominent in lead I, II, aVL, V2, V3, V5, V6

**Figure 5:**
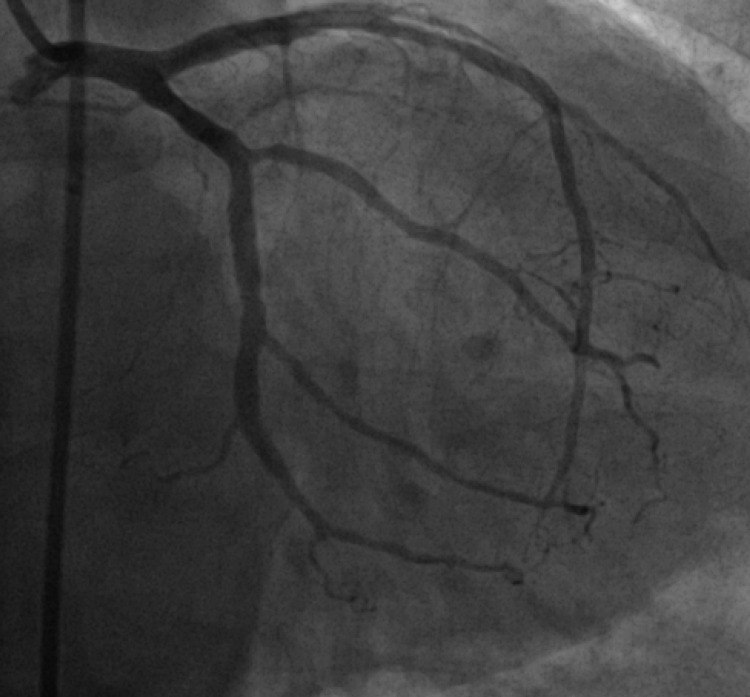
Coronary angiogram demonstrating no significant obstruction in the left coronary artery circulation Left main coronary artery was patent. Left anterior descending artery had mild luminal irregularities. Diagonal branches were patent. Left circumflex artery was codominant vessel which had mild luminal irregularities. Obtuse marginal branches were patent.

**Figure 6:**
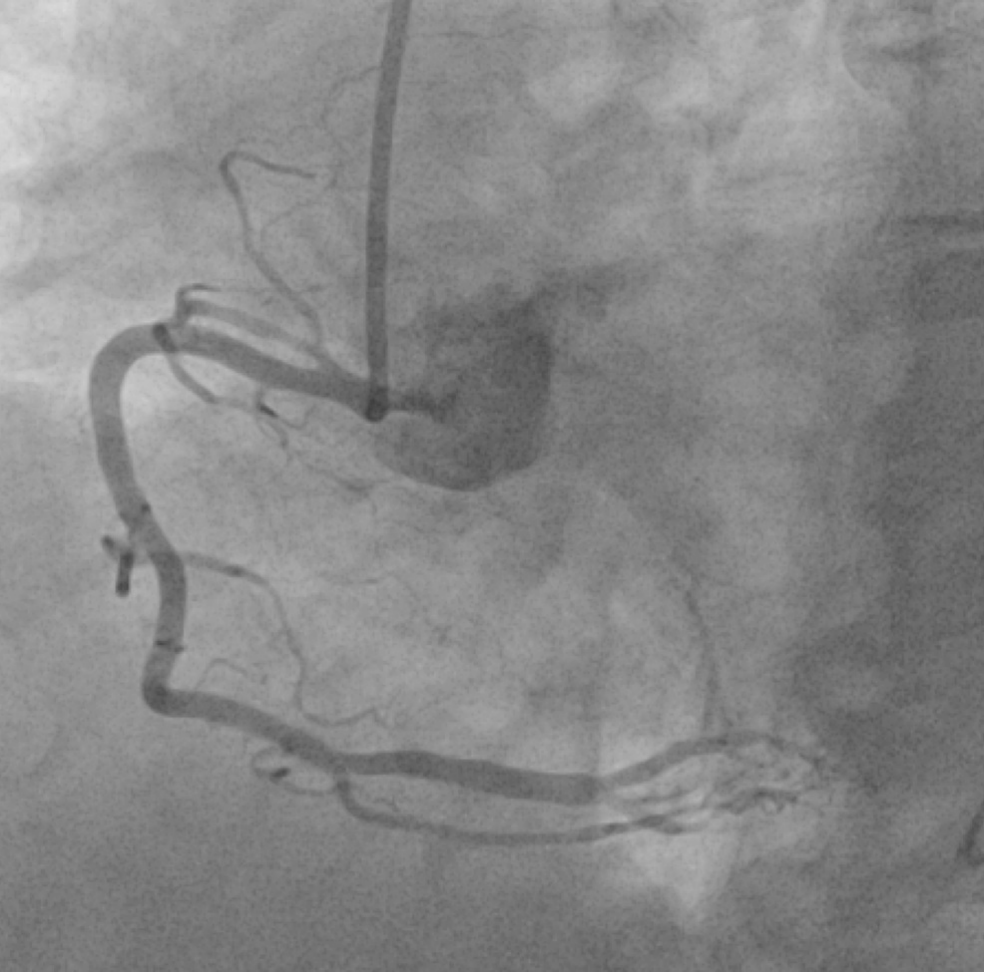
Coronary angiogram demonstrating no significant obstruction in right coronary artery circulation Right coronary artery was patent. Right posterior descending artery (PDA) and posterior-lateral branch was patent.

Overnight on hospital day 2, the patient began complaining of non-radiating, midsternal pleuritic chest pain. A repeat EKG showed ST elevation prominent in leads I, II, V2, V3, V4, V5, V6 concerning for possible post-myocardial infarction pericarditis (Figure 4). The patient underwent coronary angiogram with left heart catheterization which revealed mild non-obstructive coronary artery disease (Figures 5, 6). These findings were consistent with stress-induced cardiomyopathy.

In total, the patient was monitored over 12 days in the hospital. The patient was instructed to follow up with a cardiologist outpatient and discharged on a medical regimen of clopidogrel for three months, low-dose aspirin, beta-blocker, statin, and angiotensin-converting enzyme (ACE)-inhibitor per guideline-directed therapy. Due to high clinical suspicion of pericarditis, even though the patient didn't fully meet the criteria, she was prescribed colchicine for three months to treat pericarditis and discharged on oral vancomycin for continued use treatment of clostridium difficile colitis that developed during her hospital stay.

## Discussion

Stress-induced cardiomyopathy, or Takotsubo syndrome, is an acute dysfunction of the left ventricle myocardium that is often reversible. SIC has been most associated with physiologic states of catecholamine excess. Other proposed etiologies include metabolic disturbances, coronary microvascular impairment, and coronary artery spasm. SIC is an important diagnosis for clinicians to consider in patients presenting with cardiac complaints, new electrocardiogram findings, or cardiac biomarker elevations, as it is likely that cases of SIC go undetected and misdiagnosed. Patients with SIC usually present similar to acute coronary syndrome, with chest pain, dyspnea, or syncope. Less commonly, patients with SIC present with serious complications, including cardiogenic shock, ventricular arrhythmias, and cardiac arrest [5]. Interestingly, SIC has been described by patients as chest pressure that radiates into the neck and head, which may be the result of a rise in catecholamine levels and hypertensive surge. Patients may also present with diaphoresis and heightened sense of anxiety [2]. While those at the highest risk of SIC are post-menopausal women, it is estimated that 10% of SIC cases are men or younger women. It is also estimated that 30-35% of cases have no identifiable physical or emotional stressor preceding SIC diagnosis [3].

Since SIC is considered cardiac dysfunction in the absence of ACS, it may be considered a diagnosis of exclusion. New electrocardiogram abnormalities may be evident such as ST segment elevation, deep T wave inversions, or QT prolongation. Troponin levels may rise, but the peak troponin is typically not as high as seen in myocardial infarction, and the rise is often lower than what is expected in comparison to the amount of dysfunctional myocardium seen on ultrasound. The classic echocardiogram findings include apical ballooning of the left ventricle or hypokinesia of the cardiac apex, although multiple anatomic variants have been identified. Cardiac angiography is necessary to confirm the absence of occlusive coronary atherosclerotic disease or acute plaque rupture. Cardiac MRI would have been beneficial to investigating current diagnoses and possible diagnoses such as myocarditis given the elevated troponin revealing myocardial injury, but it was unavailable at the hospital. There is a possibility this could have been possible myopericarditis given a picture of pericarditis and myocardial injury with elevated troponin, how unable to further confirm myopericarditis given lack of cardiac MRI availability. Differential also includes hypertrophic cardiomyopathy, viral myocarditis, or any other pathological states that may be causing the transient ventricular dysfunction [2]. Patients with SIC have recovery of ventricular function on cardiac imaging typically by two months, and patients should be followed for a period of three to six months [2,6].

The patient in this case presented with non-specific symptoms of lower extremity weakness and fatigue, as well as atypical chest pains. This patient had no apparent acute physical or emotional stressor prior to the onset of her symptoms; however, she was diagnosed with and treated for clostridium difficile colitis while in the hospital. Her electrocardiogram changes were concerning for anterolateral ischemia, and the small rise seen with repeat troponin levels was discordant with the amount of hypokinesis seen on cardiac imaging. The patient was deemed high-risk for thrombotic complications and initiated on dual antiplatelet therapy. The patient was treated in adherence with protocols typically used for acute myocardial infarction while in the hospital and upon discharge.

There is currently no standard practice for long-term management after SIC. Current inpatient treatment is aimed at supportive measures and decreasing the risk of complications. Some sources recommend discontinuing the use of aspirin and P2Y12 receptor antagonists once a myocardial infarction is excluded, and others recommend continuing anticoagulation therapy until the systolic function is fully restored [2,7]. The use of beta-blockers and ACE inhibitors is considered reasonable, although there is currently limited evidence to suggest these medications improve outcomes for patients with SIC. The use of inotropes and sympathomimetics is contraindicated in SIC [2]. Further randomized controlled trials are needed to establish standardized, evidence-based medical treatment following SIC.

## Conclusions

Stress-induced cardiomyopathy is a primarily reversible dysfunction of the left ventricle myocardium which is important for clinicians to be able to differentiate from other acute cardiac syndromes. Multiple pathophysiological processes predispose patients to SIC, and treatment may vary based on clinical judgment and patient presentation. While current inpatient treatment of SIC focuses on complications and supportive therapy, further research is necessary in order to standardize long-term management.
